# Cross-Cultural Perspectives on School Absenteeism: Comparative Profiles from Italy, Spain, and Turkey

**DOI:** 10.5334/cie.238

**Published:** 2026-06-19

**Authors:** Carolina Gonzálvez, María Pérez-Marco, Andrea Fuster, Ricardo Sanmmartín, Caner Sunay, Concettina Caparello, Fatmanur Çimen, Pina Filipello, Carmelo Francesco Meduri, Ismail Seçer, Luana Sorrenti, Eda Tatli

**Affiliations:** 1 Department of Developmental Psychology and Didactics, University of Alicante, Spain; 2 Ministry of National Education, Erzurum, Türkiye; 3 Universita degli Studi di Enna ‘Kore’, Italy; 4 Ataturk University, Erzurum, Türkiye; 5 Developmental and Educational Psychology, University of Messina, Italy; 6 The University Magna Graecia of Catanzaro, Italy; 7 Guidance and Psychological Counseling Department, Ataturk University, Erzurum, Türkiye; 8 Department of Guidance and Psychological Counseling Department, Ataturk University, Erzurum, Türkiye

**Keywords:** school absenteeism, adolescents profiles, Italy, Spain, Turkey, cross-cultural perspective

## Abstract

The assessment of the school absenteeism in the world highlights a concerning situation in Southern Europe in terms of attendance problems, particularly in Italy (60.8%), Spain (28.3%), and Turkey (53.2%). These differences underscore the importance of cross-country comparisons, as prevalence rates vary due to cultural, geographical, and methodological factors given the large number of parameters that are used in each country to measure these school attendance problems. Consequently, this study investigates school absenteeism profiles across Italy, Spain and Turkey using the ARSNA instrument, focusing on truancy (TR) and school refusal (SR) dimensions. Four distinct profiles were identified: *Non-school absenteeism, Truancy, School refusal* and *Mixed school absenteeism* profiles. Cross-national differences were observed, with the *Truancy* profile being more prevalent in Turkey and the *School refusal* profile in Spain, although these differences were not statistically significant. Gender differences were only significant in Italy, where girls showed higher proportions in School Refusal-related profiles. These findings contribute to a better understanding of cross-cultural patterns of school absenteeism and may inform the development of targeted interventions.

School attendance is essential for academic success, social development and emotional well-being, providing the foundation for students’ growth (Keppens, 2023; Rogers et al., 2024). However, School Attendance Problems (SAP), including chronic absenteeism, truancy, or school refusal, remain significant challenges globally given their impact on the child and youth population (Johnsen et al., 2024). These consequences manifest both in the short term (e.g., low academic performance, difficulties in the teaching-learning process) and in the long term (e.g., reduced socio-occupational opportunities). In this study, the terms School Attendance Problems and School Absenteeism are used as umbrella and synonym constructs encompassing different forms of problematic school attendance. These issues cover the whole spectrum of problematic school attendance, clarifying this broad construct (Heyne et al., 2019). Moreover, they often result from a complex interaction of personal, familial and school-related factors, disproportionately affecting vulnerable groups (Gubbels et al., 2019; Hilwerling et al., 2022; Kim & Gentle-Genitty, 2020). School absenteeism has been shown to disrupt learning, social integration and long-term academic outcomes (Gonzálvez et al., 2023; Kearney et al., 2019; Maynard et al., 2018).

As multi-level strategies to address SAPs continue to gain prominence, the need for reliable assessment tools has been increasingly recognized (Gonzálvez et al., 2021). Among these, *School Refusal Assessment Scale-Revised* (SRAS-R; Kearney, 2002) is the most widely used instrument, having been validated in numerous countries, including Chile, Germany, Italy, the Netherlands, Spain, the United Kingdom, the United States and Turkey. However, given the diversity of factors underlying SAPs, newer instruments have been developed to address reasons not encompassed by the *School Refusal Assessment Scale-Revised* (SRAS-R; Kearney, 2002) (Gonzálvez et al., 2021). One such tool is the *Assessing Reasons for School Non-Attendance* (ARSNA; Havik et al., 2015), which was designed in Norway for 6^th^ to 10^th^ grade students. This instrument assesses four usual reasons for school absenteeism: Somatic Symptoms, Subjective Health Complaints, Truancy, and School Refusal.

Through the combination of high and low scores across the four dimensions of the SRAS-R, distinct profiles of school refusal behaviour have been identified (Dube & Orpinas, 2009; Gallé-Tessonneau et al., 2019; Gonzálvez, Inglés, Fernández-Sogorb et al., 2018; Gonzálvez, Inglés, Kearney et al., 2019; Gonzálvez, Inglés, Sanmartín et al., 2018; Gonzálvez, Kearney et al., 2018; Knollmann et al., 2013; Pérez-Marco et al., 2024). Although the ARSNA has only been validated in Spain, demonstrating a four-factor structure and adequate internal consistency (Martínez-Torres et al., 2024), its potential for identifying school absenteeism profiles remains largely unexplored.

School Refusal (SR) and Truancy (TR) are among the most widely studied and prevalent forms of School Attendance Problems (SAPs) in the fields of education and psychology (Johnsen et al., 2024; Kearney et al., 2022). SR refers to difficulties attending school associated with emotional distress, whereas TR is characterized by unauthorized and often concealed absences from school (Heyne et al., 2019). These two dimensions represent conceptually distinct patterns of absenteeism, frequently associated with different underlying mechanisms (e.g., anxiety-related avoidance vs. rule-breaking behaviour), making them particularly relevant for both research and intervention.

In the present study, the analysis focused specifically on the SR and TR dimensions of the ARSNA instrument, as they capture the most theoretically differentiated and empirically supported forms of school absenteeism across contexts (Gonzálvez et al., 2021). This decision also allows for greater comparability across countries, given that these dimensions have been more consistently examined in previous international research.

This focus is particularly relevant in light of recent international evidence. According to the latest PISA report (OECD, 2023), school attendance problems show substantial cross-national variability, with Southern European countries presenting especially concerning rates of absenteeism (e.g., Italy: 60.8%; Spain: 28.3%; Turkey: 53.2%), in contrast to more favourable patterns observed in Northern Europe. In this context, examining SR and TR provides a useful framework for understanding key differences in absenteeism profiles across countries with differing prevalence rates and socio-educational contexts. However, it is important to note that other dimensions of school attendance problems included in the full ARSNA instrument were not considered in this study. As a result, the identified profiles should be interpreted as reflecting patterns primarily driven by SR and TR.

The role of age and gender in SR and TR remains inconclusive in the existing literature (García-Fernández et al., 2016). Regarding age, some studies have reported higher prevalence rates in early adolescence (13–14 years; Kearney et al., 2006; Tolin et al., 2009), whereas others have found increased rates among older adolescents (15 years and above; García-Fernández et al., 2016; Maynard et al., 2017).

Similarly, findings related to gender are inconsistent. In the case of SR, some studies report a relatively balanced distribution between boys and girls (Fremont, 2003; Guare & Cooper, 2003; Wijetunge & Lakmini, 2011), while others indicate a higher prevalence among girls, often linked to anxiety-related factors (Fan et al., 2008; García-Fernández et al., 2016; Gosch et al., 2012). In contrast, TR has been more frequently associated with boys, particularly due to its connection with school disengagement and dropout (García-Fernández et al., 2016; Kearney, 2008; Kearney & Spears, 2014), although some studies suggest higher prevalence among girls (Maynard et al., 2017).

Moreover, much of this evidence is based on studies conducted over a decade ago, highlighting the need for updated research that reflects current social and educational contexts. Given these inconsistencies, further research is required to clarify the role of age and gender in different forms of school absenteeism, particularly within cross-cultural contexts.

## The Present Study

Given the alarming situation in Southern Europe, more cross-cultural research is needed to shed light on possible similarities, despite cultural and geographical differences. In this sense, the present study aims to, firstly, compare the different profiles of school absenteeism based solely on the two most common reasons (TR and SR) between three Southern European countries: Italy, Spain and Turkey. While theoretical evidence for ARSNA profiles has not been previously established, research using the SRAS-R has identified that the most prevalent profiles included uniformingly high or low scores across all dimensions, as well as combinations of varying scores across different factors (Dube & Orpinas, 2009; Gallé-Tessonneau et al., 2019; Gonzálvez, Inglés, Fernández-Sogorb et al., 2018; Gonzálvez, Inglés, Kearney et al., 2019; Gonzálvez, Inglés, Sanmartín et al., 2018; Gonzálvez, Kearney et al., 2018; Knollmann et al., 2013; Pérez-Marco et al., 2024). Accordingly, four distinct school absenteeism profile are expected: 1) Non-school absenteeism profile characterized by low scores in TR and SR factors; 2) Truancy profile reflecting elevated score in TR and low in SR; 3) School refusal profile with low scores in TR and high in SR; and 4) Mixed school absenteeism profile featuring a combination of high scores across both ARSNA dimensions (TR and SR). Finally, another aim is, secondly, to establish statistically significant differences between the profiles of each country in terms of age and gender, expecting not to find statistically significant differences in both gender and age, given the controversy in previous literature in TR and SR (Fan et al., 2008; Fremont, 2003; García-Fernández et al., 2016; Gosch et al., 2012; Guare & Cooper, 2003; Kearney, 2008; Kearney et al., 2006; Kearney & Spears, 2014; Maynard et al., 2017; Tolin et al., 2009; Wijetunge & Lakmini 2011).

## Method

### Participants

A random cluster sampling was used to choose participants in three countries: Italy, Spain and Turkey. Three sampling units were established in each country: a geographical area in each country, the schools in each area, and, finally, the students in each school. The final sample consisted of 1589 participants aged 14–18 years, being 539 from Italy (222 boys, 304 girls, 13 other; *M* = 16.05, *SD* = .99), 531 from Spain (225 boys, 295 girls, 11 other; *M* = 15.66, *SD* =1.27), and 519 from Turkey (348 boys, 171 girls, *M* = 15.98, *SD* = 1.14). The sample was homogeneously distributed by gender and age, as no statistically significant differences were found for gender and age in Italy (*χ^2^* = 13.95; *p* = .08), in Spain (*χ^2^* = 5.18; *p* = .74), and in Turkey (*χ^2^* = 8.81; *p* = .07).

### Instrument

*Assessing Reasons for School Non-attendance* (ARSNA; Havik et al., 2015). Using a 4-point Likert scale (0 = never, 1 = rarely, 2 = occasionally, and 3 = very often), students’ reasons for missing school were assessed using this 17-item self-report instrument. Each item was framed around the question, “How often have you been absent from school in the last three months because…”. Items cover four dimensions: *Somatic Symptoms* (SS) (“…you had a bad cold or flu?); *Subjective Health Complaints* (HC) (“…you had a headache?”); *Truancy* (TR) (“…you had a meeting with friends?”); and *School Refusal* (SR) (“…you wanted to avoid unpleasant situations at school?”). For the present study, only the dimensions of TR (items 10, 11, 12 and 13) and SR (items 14, 15, 16 and 17) were used.

Given the cross-national nature of this study, particular attention was paid to the comparability of the measurement instrument across countries. The ARSNA scale has been previously validated in the Spanish context (Martínez-Torres et al., 2024). For the Italian and Turkish samples, the instrument was adapted following the International Test Commission Guidelines for Translating and Adapting Tests (International Test Commission, 2017). A rigorous back-translation procedure was implemented to ensure semantic and conceptual equivalence across languages. Specifically, two bilingual experts in Educational Sciences and Psychology from each country independently translated and reviewed the instrument. The resulting versions were evaluated for cultural and contextual appropriateness, and no substantial modifications were required. Expert evaluators confirmed that the items were clear, appropriate, and suitable for use in each context. In addition, the internal consistency of the TR and SR dimensions was found to be acceptable across countries (Italy: α = .69 and .80; Spain: α = .76 and .78; Turkey: α = .74 and .77), supporting the reliability of the measures. Although reliability indices in Italy were slightly lower in TR and approached the commonly accepted threshold, they can still be considered adequate for research purposes. Overall, these results support the use of the selected dimensions in the present cross-national analysis, while suggesting that findings should be interpreted with some caution in contexts where reliability is comparatively lower. However, it should be noted that formal measurement invariance across countries was not tested. Therefore, although the instrument was carefully adapted and showed adequate reliability, cross-country comparisons should be interpreted with caution, as potential cultural and linguistic differences may influence the comparability of the results.

### Procedure

In each participating country, a meeting was conducted to present the study objectives to the school councils of the selected institutions. Furthermore, parents and/or legal guardians provided signed and written informed consent for their children’s participation. Students completed self-report questionnaires during school hours, with confidentiality and voluntary participation assured. To ensure consistency in the administration process, a researcher was present in the classroom throughout the 20-minute assessment period. The study also received ethical approval from the Ethics Committee of the University of Alicante (UA-2023-03-07).

### Data Analysis

A non-hierarchical cluster analysis (quick cluster analyses) was conducted to distinguish between school absenteeism profiles. In contrast to hierarchical methods, non-hierarchical approaches enable the number of desired groups to be specified in advance, providing a single cluster solution. Additionally, the method allows individual initially assigned to one group to be reassigned to another at a later stage if doing so improves the selection criterion (Clatworthy et al., 2005). Quick cluster analysis is also regarded as the most suitable technique for identifying profiles within large samples of participants (Hair et al., 1998). Prior to the cluster analysis, the data were standardized for each ARSNA dimension (TR and SR) separately. Results were interpreted using *z-*scores, with values below – .5, between – .5 and + .5, and above + .5 classified as low, moderate, and high levels of TR and SR, respectively. Moreover, a Chi-squared test of homogeneity was applied to examine differences in the frequency distribution of participants across clusters based on gender, age, and the overall sample in each country separately and between countries. When significant results were obtained, differences between categories were interpreted through comparisons of proportions across groups. Statistically significant differences were determined using *p*-values below .05 (Cohen, 1988). IBM SPSS Statistics version 28 was used for the statistical analysis.

## Results

### Difference in Proportions of Subjects Classified in Each Cluster in Each Country

**Figure 1:**
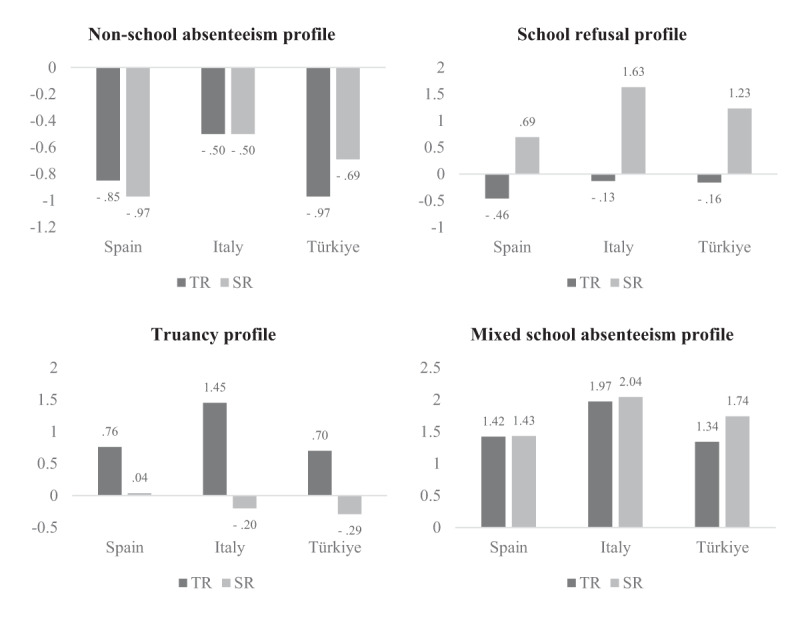
School absenteeism profiles in each country.

Figure 1 showed the four-cluster model for School Absenteeism based on the combination of punctuations of TR and SR in each country. The first profile was *Non-school absenteeism* profile with low scores in each factor, TR and SR (Spain: –.85, –.97; Italy: –.50, –.50; Turkey: –.97, –.69, respectively), including 208 students in Spain, 358 in Italy and 202 in Turkey. Secondly, the *School refusal* profile scored low levels of TR and high levels of SR in each country (Spain: –.46, .69; Italy: –.13, 1.63; Turkey: –.16, 1.23, respectively), with 104 students in Spain, 66 in Italy and 74 in Turkey. The third profile was *Truancy* profile with high scores in TR and low in SR (Spain: .76, .04; Italy: 1.45, –.2; Turkey: .7, –.29, respectively), including 131 students in Spain, 78 in Italy and 184 in Turkey. The fourth profile was *Mixed school absenteeism* due to high scores in both TR and SR factors (Spain: 1.42, 1.43; Italy: 1.97, 2.04; Turkey: 1.34, 1.74, respectively), incorporating 88 students in Spain, 37 in Italy and 59 in Turkey. Finally, to facilitate the interpretation of the identified clusters, Table 1 provides a conceptual summary of the main characteristics associated with each school absenteeism profile.

**Table 1:** Conceptual description of School Absenteeism profiles.


PROFILES	SR SCORES	TR SCORES	DESCRIPTION

Non-school absenteeism (NSA)	Low	Low	Students with low levels of both school refusal and truancy behaviours, indicating generally regular school attendance.

School refusal (SR)	High	Low	Students showing elevated emotional distress-related absenteeism, typically linked to anxiety or school-related fear.

Truancy (TR)	Low	High	Students characterized by unauthorized absences, often associated with disengagement or rule-breaking behaviours.

Mixed school absenteeism (MSA)	High	High	Students presenting both emotional distress-related avoidance and truancy behaviours, reflecting a more complex absenteeism profile.


Chi-squared test of homogeneity showed statistically significant differences between each cluster in each country: Spain (*χ^2^* (3) = 63.99; *p* < .001), Italy (*χ^2^* (3) = 499.76; *p* < .001) and Turkey (*χ^2^*(3) = 125.45; *p* < .001), suggesting a heterogeneous distribution of profiles in all cases. Furthermore, Table 2 indicates the differences in proportion of subjects classified in each profile between Spain, Italy and Turkey. To examine cross-country differences, pairwise comparisons of proportions were conducted. Statistically significant differences were observed across countries for all profiles. Specifically, the *Non-school absenteeism* profile was significantly more prevalent in Italy compared to Spain and Turkey. The *School refusal* profile was significantly more frequent in Spain than in Italy and Turkey. The *Truancy* profile showed significantly higher proportions in Turkey compared to Spain and Italy, and was also higher in Spain than in Italy. Finally, the *Mixed school absenteeism* profile was significantly more prevalent in Spain than in Turkey and Italy, and higher in Turkey than in Italy.

**Table 2:** Difference in proportions of subjects classified in each cluster between countries.


	SPAIN (N_TOTAL_ = 531)	ITALY (N_TOTAL_ = 539)	TURKEY (N_TOTAL_ = 519)	STATISTICAL SIGNIFICANCE

NSA	208 39.1%	358 66.4%	202 38.9%	Italy > Spain Italy > Turkey

SR	104 19.6%	66 12.2%	74 14.3%	Spain > Italy Spain > Turkey

TR	131 24.7%	78 14.5%	184 35.5%	Turkey > Spain > Italy

MSA	88 16.6%	37 6.9%	59 11.4%	Spain > Turkey > Italy


*Note*. NSA = Non-school absenteeism profile; SR = School refusal profile; TR = Truancy profile; MSA = Mixed school absenteeism profile. Percentages may not sum to 100 due to rounding.

### Difference in Proportions in Each Cluster by Gender and Age for Each Country

In terms of gender, Table 3 showed the differences in proportions in each profile for each country. Gender differences in school absenteeism profiles were examined using chi-square tests within each country. Statistically significant differences were found only in Italy (*χ^2^*(3) = 32.54, *p* < .001). In this case, while girls were significantly higher on the *Non-school absenteeism, School refusal* and *Mixed school absenteeism* profiles, boys were only significantly more represented in the *Truancy* profile.

**Table 3:** Differences in proportions in each cluster by gender for each country.


		NSA	SR	TR	MSA	TOTAL GENDER	*χ^2^(df), p*

Spain (*N* = 520)	Boys	79 35.1%	39 17.3%	64 28.4%	43 19.1%	225 100%	*χ^2^*(3) = 7.14 *p* = .07

	Girls	127 43.1%	62 21%	63 21.4%	43 14.6%	295 100%	

Italy (*N* = 526)	Boys	157 70.7%	9 4.1%	44 19.8%	12 5.4%	222 100%	*χ^2^*(3) = 32.54 *p* < .001

	Girls	194 63.8%	56 18.4%	30 9.9%	24 7.9%	304 100%	

Turkey (*N* = 519)	Boys	141 40.5%	48 13.8%	115 33%	44 12.6%	348 100%	*χ^2^*(3) = 4.09 *p* = .25

	Girls	61 35.7%	26 15.2%	69 40.4%	15 8.8%	171 100%	


*Note*. NSA = Non-school absenteeism profile; SR = School refusal profile; TR = Truancy profile; MSA = Mixed school absenteeism profile. Percentages may not sum to 100 due to rounding.

In contrast, no statistically significant gender differences were found in Spain (*χ^2^*(3) = 7.14, *p* = .07) or Turkey (*χ^2^*(3) = 4.09, *p* = .25), although some descriptive differences were observed. In Spain, the *Non-school absenteeism* and *School refusal* profiles showed slightly higher proportions of girls, while the *Truancy* profile had a higher number of boys as well, but not significantly. However, in Turkey, boys showed marginally higher proportions across all profiles. Finally, it should be noted that participants reporting “Other” gender (Spain = 11; Italy = 13) were excluded from gender-based analyses to ensure comparability due to the dual distinction of boy and girl of Turkey. The analytic sample for these analyses was Spain (*N* = 520), Italy (*N* = 526), and Turkey (*N* = 519).

In terms of age, Table 4 shows the differences in the proportions of each profile according to age in each country. No statistically significant differences were found for age in Spain (*χ^2^*(12) = 19.71, *p* = .07), Italy (*χ^2^*(12) = 13.85, *p* = .31), or Turkey (*χ^2^*(12) = 10.01, *p* = .62). Although no significant differences were observed, some descriptive patterns emerged. In Spain, the *Non-school absenteeism* profile showed higher proportions among students aged 16 and 17, while the *School refusal* profile was relatively evenly distributed across age groups, with slightly higher values at ages 16 and 18. The *Truancy* profile showed a somewhat higher proportion among 15- and 16-year-old students, whereas the *Mixed school absenteeism* profile was more frequent among younger students, particularly those aged 14 and 15. In Italy, the *Non-school absenteeism* profile predominated across all age groups, especially among students aged 15 and 16. The *School refusal* and *Truancy* profiles showed relatively similar distributions across ages, with slightly higher proportions in mid-adolescence (15–17 years). The *Mixed school absenteeism* profile remained comparatively low across all age groups. In Turkey, the distribution of profiles was relatively stable across age groups. The *Non-school absenteeism* profile showed similar proportions across ages, while the *Truancy* profile tended to be slightly more frequent among students aged 15 to 18. The *School refusal* and *Mixed school absenteeism* profiles showed no clear age-related pattern. It is also important to note that, in Italy, 13 participants were excluded from this analysis because they were outside the target age range (14–18 years), in order to ensure comparability across countries.

**Table 4:** Differences in proportions in each cluster by age for each country.


		NSA	SR	TR	MSA	TOTAL AGE	*χ^2^(df), p*

Spain (*N* = 520)	14	43 34.7%	23 18.5%	25 20.2%	33 26.6%	124 100%	*χ^2^*(12) = 19.71 *p* = .07

	15	38 33.3%	22 19.3%	34 29.8%	20 17.5%	114 100%	

	16	60 45.1%	25 18.8%	34 25.6%	14 10.5%	133 100%	

	17	47 45.6%	20 19.4%	22 21.4%	14 13.6%	103 100%	

	18	17 37%	12 26.1%	12 26.1%	5 10.9%	46 100%	

Italy (*N* = 526)	14	15 83.3%	3 16.7%	0 0%	0 0%	18 100%	*χ^2^*(12) = 13.85 *p* = .31

	15	108 69.7%	17 11%	20 12.9%	10 6.5%	155 100%	

	16	117 69.2%	21 12.4%	23 13.6%	8 4.7%	169 100%	

	17	97 61.4%	21 13.3%	24 15.2%	16 10.1%	158 100%	

	18	14 53.8%	3 11.5%	7 26.9%	2 7.7%	26 100%	

Turkey (*N* = 519)	14	17 39.5%	7 16.3%	12 27.9%	7 16.3%	43 100%	*χ^2^*(12) = 10.01 *p* = .62

	15	68 43.9%	21 13.5%	56 36.1%	10 6.5%	155 100%	

	16	59 39.9%	19 12.8%	50 33.8%	20 13.5%	148 100%	

	17	39 34.2%	17 14.9%	42 36.8%	16 14%	114 100%	

	18	19 32.2%	10 16.9%	24 40.7%	6 10.2%	59 100%	


*Note*. NSA = Non-school absenteeism profile; SR = School refusal profile; TR = Truancy profile; MSA = Mixed school absenteeism profile. Percentages may not sum to 100 due to rounding.

## Discussion

The present study aims to compare the different profiles of school absenteeism based solely on the two most common reasons of non-attendance (TR and SR) between Italy, Spain and Turkey, as well as to analyse the possible statistically significant differences between profiles in terms of age and gender among the three countries. Firstly, the cluster analyses demonstrated the suitability of the 4-profile model based on the two most prevalent ARSNA dimensions (TR and SR), resulting in the following profiles: *Non-school absenteeism, School refusal, Truancy* and *Mixed school absenteeism* profiles. Therefore, the first hypothesis was fully supported, given the coincidence with the number and the type of profiles found in line with the previous literature (Dube & Orpinas, 2009; Gallé-Tessonneau et al., 2019; Gonzálvez, Inglés, Fernández-Sogorb et al., 2018; Gonzálvez, Inglés, Kearney et al., 2019; Gonzálvez, Inglés, Sanmartín et al., 2018; Gonzálvez, Kearney et al., 2018; Knollmann et al., 2013; Pérez-Marco et al., 2024).

These differences likely reflect the complex interplay of cultural, educational, and socio-institutional factors across Italy, Spain, and Turkey. Rather than being attributable to a single cause, the observed patterns may be related to differences in how school attendance is monitored, how absenteeism is socially perceived, and how emotional and behavioural difficulties are identified and managed within each educational system (Gubbels et al., 2019).

For example, the higher prevalence of the *Truancy* profile in Turkey may be related to differences in attendance monitoring practices, school engagement strategies, or broader socio-educational dynamics that influence student–school relationships. In contrast, the relatively higher presence of the *School refusal* profile in Spain may be associated with a greater awareness and identification of emotional distress in school settings, as well as increased access to psychological support services. In Italy, the predominance of the *Non-school absenteeism* profile may suggest a comparatively lower prevalence of behaviourally or emotionally driven school attendance problem within the sampled population, although this interpretation should be considered cautiously given potential contextual and measurement differences.

Regarding the differences in terms of gender and age, the hypothesis was partially confirmed (Fan et al., 2008; Fremont, 2003; García-Fernández et al., 2016; Gosch et al., 2012; Guare & Cooper, 2003; Kearney, 2008; Kearney et al., 2006; Kearney & Spear, 2014; Maynard et al., 2017; Tolin et al., 2009; Wijetunge & Lakmini 2011). Despite the predominance of non-statistical significance in both, it was found only that, in Italy, girls scored statistically higher in the *Non-school absenteeism, School refusal* and *Mixed school absenteeism* profiles; whereas boys were significantly more prevalent in the *Truancy* profile. In line with Italy, Spain is reported to show the same gender pattern, but without being statistically significant. Moreover, these gender differences, where girls are more likely to exhibit *Non-school absenteeism, School refusal* and *Mixed school absenteeism* profiles may be linked to higher anxiety levels among females, as noted in previous research (Fan et al., 2008; García-Fernández et al., 2016; Gosch et al., 2012) and the prevalence of emotionally based school non-attendance problems among girls (Corcoran et al., 2024). However, in the case of Turkey, boys predominate in the conformation of all the profiles found, which may be due to the prevalence of boys in the total sample from this country.

With regard to age, no statistical significance was found for any country or for any age group. However, it has been observed that in both Spain and Turkey students aged 15 are the majority in the *Non-school absenteeism, School refusal* and *Truancy* profiles; while in Italy, the average age of participants in each profile is higher between 16 and 17 years, in line with previous literature (García-Fernández et al., 2016; Maynard et al., 2017). Therefore, the older the student, the more school attendance problems are observed.

### Implication for Practice

This cross-cultural research provides valuable insights for the development of interventions to address school attendance problems globally. By identifying different school non-attendance profiles, educators and policy makers can design culturally sensitive strategies tailored to the specific needs of each profile. For example, interventions focused on emotional support and anxiety reduction may benefit students with the School refusal profile, while those with the *truancy* profile may require stricter attendance monitoring and engagement strategies (Pérez-Marco, Gonzálvez et al., 2025).

Given the focus on supporting continuity in education, the findings of this study may have important implications for students with medical or mental health conditions. While the profiles identified reflect general patterns of school absenteeism, they may also inform the identification of students whose non-attendance is linked to chronic illness or clinically significant anxiety. For example, students exhibiting a *School refusal* profile may benefit from interventions that address emotional distress, anxiety management, and supportive re-entry strategies, whereas students in the *Truancy* or *Mixed school absenteeism* profiles may require tailored engagement plans alongside monitoring to differentiate health-related absences from other forms of non-attendance (Pérez-Marco, Gonzálvez et al., 2025). Practical applications might include flexible timetables, planned re-entry programs, catch-up or remote bridging strategies, named staff contacts, and linkage with healthcare plans (Capurso et al., 2025; Irwin et al., 2024). These considerations can help prevent the misclassification of health or anxiety-related absence as truancy and promote continuity in education, while acknowledging that the findings are based on a general adolescent sample and, they should be cautiously tailored to specific individuals, contexts, and cultures (Capurso et al., 2025).

Finally, the results also underline the importance of fostering a positive school climate, as it can mitigate absenteeism by promoting a sense of belonging and emotional security (Cunningham et al., 2022; Datu et al., 2023; Pérez-Marco, Fuster et al., 2025; Tomberli & Ciucci, 2021). Cross-cultural studies such as this one, highlight the need for international collaboration to address common educational challenges and improve attendance outcomes in diverse contexts, as well as to pool efforts to homogenise SAP assessment parameters, thus facilitating comparability and universality of intervention programs.

## Conclusion

Overall, this study contributes to a better understanding of school attendance problems from a cross-cultural perspective, highlighting both shared patterns and contextual differences (Heyne & Brouwer-Borghuis, 2022). These findings support the need for flexible, context-sensitive approaches to assessment and intervention, as well as continued international research to refine the conceptualization and measurement of school attendance problems.

### Limits of the Study

Despite its contributions, this study has several limitations that should be underlined. First, the sample is restricted to adolescents aged 14–18, which limits the generalisation of the findings to other age groups. Expanding the age sample to include younger students might provide a more comprehensive understanding of school attendance problems. Second, the study relies on self-reported data, which may introduce social desirability bias and affect the accuracy of the responses (Fernández-Montalvo & Echeburúa, 2006). Additionally, the cross-sectional design prevents the establishment of causal relationships between school absenteeism and its contributing factors. Longitudinal design would be valued to explore changes in absenteeism profiles over time and identify causal pathways.

Although the ARSNA instrument has been validated in Spain, its use in Italy and Turkey relied on careful translation and adaptation procedures. However, measurement invariance across countries was not formally tested, which may limit the comparability of the findings across cultural contexts, which should be addressed in the future research. An additional limitation relates to the exclusive focus on the TR and SR dimensions of the ARSNA instrument. While this decision was theoretically grounded and aimed at enhancing cross-country comparability, it may have limited the identification of other relevant patterns of school absenteeism associated with health-related or somatic factors.

## Use of Artificial Intelligence

The authors declare that Artificial Intelligence (AI) tools were used solely for language editing and grammatical correction to enhance the readability of the manuscript.
